# Self-Mutilation: A Way to Protect Yourself From a Committed Crime or to Gain Personal Benefits

**DOI:** 10.7759/cureus.48085

**Published:** 2023-10-31

**Authors:** Biliana Mileva, Metodi Goshev, Mihaela Georgieva, Ivan I Tsranchev, Alexandar Alexandrov

**Affiliations:** 1 Department of Forensic Medicine and Deontology, Medical University Sofia, Sofia, BGR; 2 Department of Forensic Medicine and Deontology, Medical University of Plovdiv, Plovdiv, BGR

**Keywords:** intimate partner violence (ipv), injury pattern, artificial injuries, clinical forensic medicine, self-harm, self-inflicted injuries

## Abstract

Self-mutilation refers to the state in which a person deliberately hurts himself without the intention to commit suicide but with the motive of some personal gain. Four cases are described in the current study with four different personal motives - drug supply, accusation of intimate partner violence, confrontation of parental prohibition, and a way to hide and escape from a committed crime. Evaluating the injuries due to self-mutilation might be challenging due to atypical lesions and well-structured false stories when the victim has some level of competency. Careful analysis of the victim’s story and a thorough evaluation of the sustained injuries are needed. If doubts about artificial injuries exist, immediate consultation with a forensic pathologist is required.

## Introduction

Clinical forensic medicine deals with victims of different types of assaults and accidents. The forensic pathologist, forensic nurse, or any other physician examining a victim should scrutinize the information and the morphology of the inflicted injuries. The medical professional involved in the case should be able to conclude about the kind of the sustained injuries - abrasions, bruises, lacerations, stab wounds, incised wounds, etc., the mechanism of the injury, and if it corresponds to the given information and the age of the lesions [1]. Additional forensic examination is often needed to distinguish self-inflicted injuries from those inflicted by another person [2,3].

Self-mutilation is not uncommon in psychiatric and forensic practice and is defined as deliberate harm to the body without intending suicide [4]. Self-inflicted injuries are usually motivated by some form of personal gain [5] or they are a sign of a severe underlying psychiatric illness [4].

In the present study, we would like to present four cases of self-inflicted injuries with four different motives to describe the putative story, the morphology of the wounds, and the confession/revealing of the truth.

## Case presentation

Case 1

A man in his 20s was arrested for 72 hours. He was known as a drug abuser. Approximately 10 hours before his release, he destroyed a lighter (kept hidden in his clothes), took the metal part, and cut both his wrists with it. He said to the police officer that he wanted to commit suicide. Immediately, physicians from the emergency department and a forensic pathologist were called to examine the person and describe the sustained injuries.

Forensic Examination

**Figure 1:**
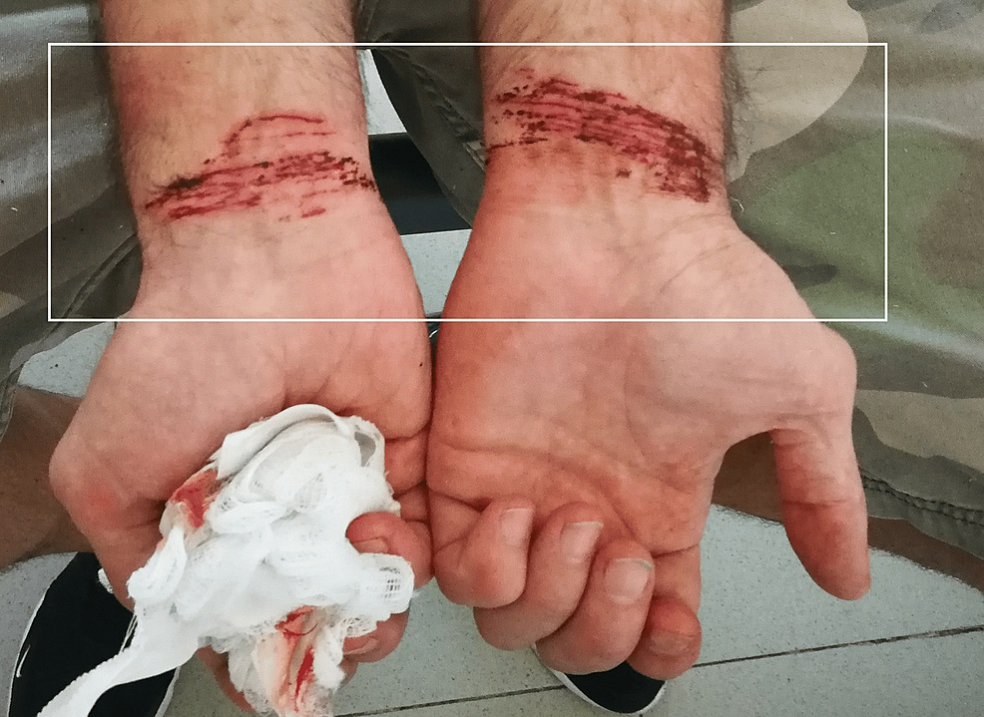
Multiple, parallel superficial self-inflicted abrasions

A forensic examination was performed following the examination of the physicians. The patient was calm and relaxed. When asked why he wanted to commit suicide, he claimed: “Doctor, I am not crazy. I didn’t want to commit suicide, I just wanted to injure myself to obtain medications from the other doctors. They gave me Diazepam.” On the palmar surfaces of both of his wrists, there were multiple parallel to each other abrasions, ranging from superficial to deep ones with minor oozing of blood. It was obvious that more injuries were inflicted on his left wrist (his dominant hand was the right) (Figure 1). After the examinations, he was released and free to go home.

Case 2

**Figure 2:**
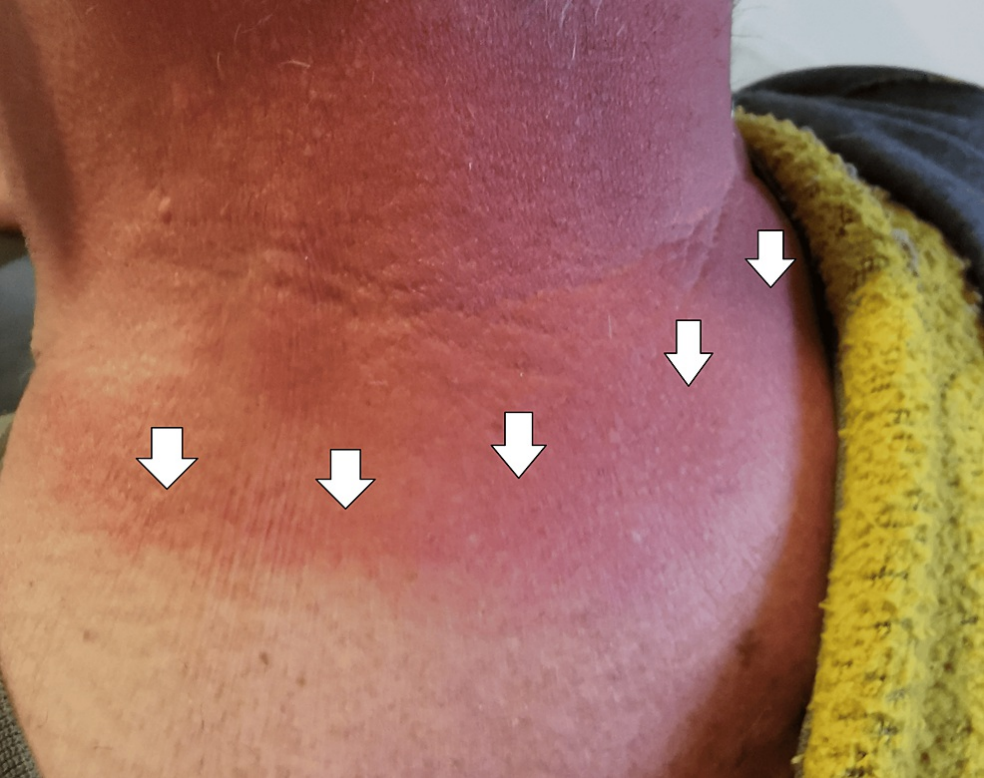
Sunburn with a distinctive line of demarcation separating the affected skin and the zones covered with clothes

A man in his 50s was examined because he said he was a victim of intimate partner violence. When asked what happened, he first said that his wife had spilled a hot coffee over his right forearm. When examined, it was noted that the whole forearm was uniformly red. There was no sharp demarcation corresponding to the limits of contact with the suspected hot fluid or trickle pattern present when the fluid runs under due to gravity. Then he was additionally asked to explain again what exactly had happened, and he changed the version. He explained that she had hit him with a hot towel, but he was unsure exactly how. Then he was asked to remove all of his clothes and a typical sunburn pattern was noted, which affected all the surfaces of his body, not covered with his t-shirt. When a finger was pressed in the areas, the zone blanched (Figure 2).

Case 3

**Figure 3:**
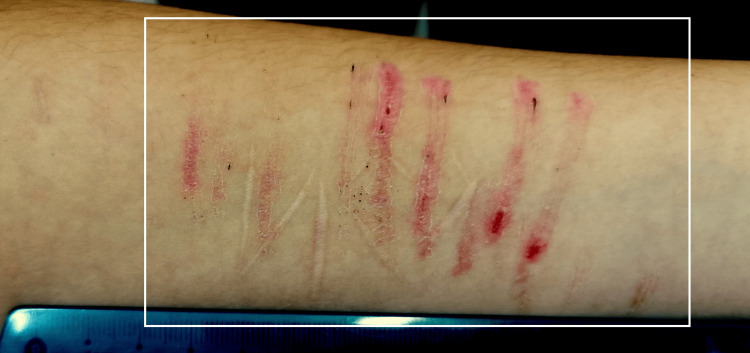
Whitish scars forming a name crossed by multiple parallel abrasions – both self-inflicted injuries

A young teenage girl was brought for a forensic examination by her parents to check if she had sexual contact. She declined to have been sexually active, and the statement was confirmed during the forensic examination. There were no signs of recent or already healed traumas in the anogenital region, and the hymen was intact, without deflorations. An additional finding was noted on the palmar surface of her left forearm. Whitish scars were forming a name written in Bulgarian - "Иси." Over this finding, there were multiple, parallel to each other, linear abrasions in a stage of healing, with fallen scabs. When asked how these injuries occurred on her forearm, she explained that she injured herself with a knife and fingernails (Figure 3). This was her boyfriend's name, and she did this to punish her parents because they didn't allow her to meet him.

Case 4

A man in his 20s was brought for a forensic examination by the police because he stated that he was a victim of physical assault and robbery during the night. When asked to describe the situation in detail, he could not do so. He said he worked in a shop for alcoholic beverages and cigarettes when the electricity was turned off. He then went to the door to check if the problem was only in his shop, and he saw a man dressed in black clothes and wearing a black mask running toward him. The man came into the shop and touched him in the cardiac area. The examined patient then stated that he fainted and hit his head on the counter in front of him. He explained that this had happened before, and he didn’t know why and started to ask the doctors if they knew why and what could cause this condition. When asked if he had some known cardiac problem, he said that he had, but he could not explain it and didn’t have medical documentation. At first, he explained that he could not hit another human being, then he changed the version and said that, for a moment, he was grasping and pushing the perpetrator. When he regained consciousness, it was because his regular client came and touched him on the leg. Then, he noticed that money was missing from the shop.

**Figure 4:**
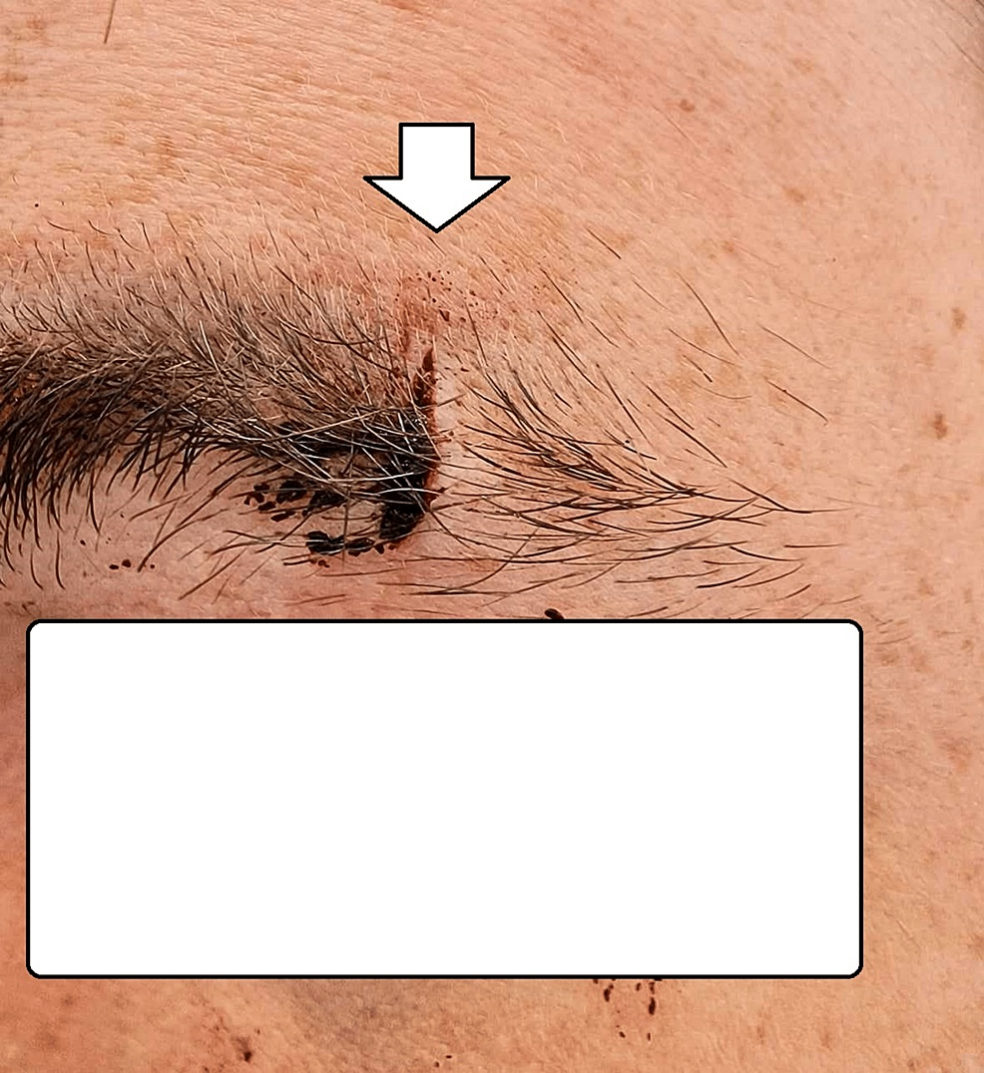
Self-inflicted incised wound on the eyebrow

On examination, a single wound of length approximately 1.5 cm was present on his left eyebrow. The wound crossed the last part of the eyebrow, was covered with hair, and was stained with dried blood. The area was carefully cleaned, and it was noted that its edges were pointed, and the margins were relatively clean-cut. The wound was superficial with a depth of approximately 1-3 mm and was surrounded with minor tissue edema. The conclusion was that the injury was an incised wound produced by the action of a sharp object and that its mechanism of occurrence did not correspond to the given information (Figure 4). Later, he confessed that he made up the story to steal the money from the shop and cut himself with a knife, watching himself in a mirror. His dominant hand was the right one.

## Discussion

Self-inflicted injuries, also known as artificial injuries [1] or self-mutilation, are a phenomenon observed in forensic and psychiatric practice [4,6]. The term mentioned means deliberate destruction of one’s body tissue without intending suicide [7]. Experts examining victims of different types of assaults or accidents should be careful and think critically about the given information since there is always a chance of the case being associated with self-inflicted injuries. A wrong interpretation of the present findings might lead to a false accusation for a crime against an innocent person or an insurance fraud [3,8,9]. Although there is a vast range of means by which a person can injure his own body, based on the presented cases in the literature, some common, repetitive features associated with self-mutilation should be known by everyone involved in the examination of an injured person [2]. Most commonly, self-inflicted injuries are incised wounds or abrasions caused by sharp or semi-sharp objects like fingernails - mechanisms of low intensity grouped in clusters of superficial lesions with similar depth, and the victim has problems explaining clearly how they occurred [1,2,4,5,10]. Typically, signs of blunt force traumas are missing, or if present, they are discreet due to the low intensity of the applied force [10]. There are no signs of struggle and defense wounds [10]. Another typical feature is that the sustained injuries are superficial, not life-threatening, and are localized in easily accessible parts of the human body, usually the forehead, cheeks, neck, chest, abdomen, anterior thighs, and forearms, or opposite side of the dominant hand, and avoiding sensitive areas such as the lips, nipples, and genitals [1,2,4-6,9-11]. Healed scars of previous self-harming actions might be present over the body [1]. Typically, the clothing is removed and unaffected by the causative object [1,2,4,9-11]. One of the most significant features is the symmetry of the injuries - similar lesions are made at the opposite limbs, “mirror images” [1,4]. Sometimes, the injuries might be performed with the help of a mirror so the final appearance of the wound might be reversed, as in the case presented by Winskog [11]. Another typical finding is the so-called “chessboard” pattern of the injury, as reported by Byard; this is a cluster of incised wounds that run parallel and at right angles to each other, creating a series of “squares” [2,4].

The presented cases have features similar to those mentioned above. The first case had the typical appearance of self-inflicted injuries - multiple, parallel to each other, abrasions, more pronounced on the left hand since the right was the dominant one. They were all superficial and not able to cause severe health consequences. The only intention was to receive medications. The second case was not actually the typical self-inflicted injury. Still, it was similar since the patient used his current pathological condition (sunburn) to accuse his wife of intimate partner violence. The third case again has the typical features of self-mutilation - present on the palmar surface of the left forearm, old healed scars, and new, multiple, parallel to each other, abrasions in the same area. The intention was parental punishment and protest against their prohibition. The last one again was situated on the side opposite to the dominant hand - the left eyebrow - the zone was easily accessible for his hand. The type of sustained injury (sharp force trauma, incised injury) does not correspond to the given information from him - impact with the counter in front of him or blunt impact. The motive was to protect himself from the committed crime - stealing money.

There are peculiar cases reported in the literature with more severe injuries involving even fractures of the bones, malinger traffic, or work-related accidents. Low-intensity forces do not produce these injuries, so usually, they are made with the help of other people but by highly organized criminal gangs, including different people, which can reproduce different fracture patterns precisely [7,12]. Mauf et al. report a case where the “victim” was a nurse, used her knowledge to describe the needed symptoms for her injuries, and made the injuries with the help of makeup [13]. The injuries might sometimes be fabricated firearm wounds [14] or have serious health consequences and even fatal outcomes [15].

## Conclusions

We present four cases of deliberate self-harm with different motives of gain. Although relatively often seen in forensic practice, such cases must be thoroughly discussed and present in the literature to enrich it and to raise awareness among the physicians involved in examinations of injured people without the needed competency, especially in the emergency care units. When there is a doubt that the injuries do not correspond to the given information/mechanism of occurrence and are suspected to be self-inflicted, a second, more experienced opinion must be searched for.
